# Biodiversity on Broadway - Enigmatic Diversity of the Societies of Ants (Formicidae) on the Streets of New York City

**DOI:** 10.1371/journal.pone.0013222

**Published:** 2010-10-05

**Authors:** Marko Pećarević, James Danoff-Burg, Robert R. Dunn

**Affiliations:** 1 Department of Ecology, Evolution, and Environmental Biology (E3B), Columbia University in the City of New York, New York, New York, United States of America; 2 State Institute for Nature Protection, Zagreb, Croatia; 3 Center for Environment, Economy, and Society (CEES), Columbia University in the City of New York, New York, New York, United States of America; 4 Department of Biology and Keck Behavioral Biology Center, North Carolina State University, Raleigh, North Carolina, United States of America; University of Arizona, United States of America

## Abstract

Each year, a larger proportion of the Earth's surface is urbanized, and a larger proportion of the people on Earth lives in those urban areas. The everyday nature, however, that humans encounter in cities remains poorly understood. Here, we consider perhaps the most urban green habitat, street medians. We sampled ants from forty-four medians along three boulevards in New York City and examined how median properties affect the abundance and species richness of native and introduced ants found on them. Ant species richness varied among streets and increased with area but was independent of the other median attributes measured. Ant assemblages were highly nested, with three numerically dominant species present at all medians and additional species present at a subset of medians. The most common ant species were the introduced Pavement ant (*Tetramorium caespitum*) and the native Thief ant (*Solenopsis molesta*) and Cornfield ant (*Lasius neoniger*). The common introduced species on the medians responded differently to natural and disturbed elements of medians. *Tetramorium caespitum* was most abundant in small medians, with the greatest edge/area ratio, particularly if those medians had few trees, whereas *Nylanderia flavipes* was most abundant in the largest medians, particularly if they had more trees. Many of the species encountered in Manhattan were similar to those found in other large North American cities, such that a relatively small subset of ant species probably represent most of the encounters humans have with ants in North America.

## Introduction

Urban and suburban areas are increasing in number and population density globally [1], [2]. Worldwide, 2003 was the first year that more people lived in urban areas than in rural ones [3]. With these shifts, the recognition of the role of urban ecological health and conservation is increasing [3], [4], [5]. Barring apocalyptic catastrophes [e.g., 6], urban and suburban habitats will be among the most common habitats on Earth for many generations to come and the most common setting for human interactions with wild species [5]. However, urban environments vary (and have long varied) in their diversity and ecological health from complex urban gardens to habitats superficially devoid of life such as the glass walls of skyscrapers [7]. Similarly, the species found in these habitats range from pests and pathogens, to species beneficial to conservation, ecosystem health, human health or human appreciation of and interaction with nature [8], [9]. A central question then for urban ecology is to understand which species persist in urban environments and what governs their diversity and composition.

Reviews emphasize that urban ecology has become an increasing area of research [e.g. 10], [11], [12]. However, we still better understand more “natural” landscape elements within urban habitats than we do constructed elements such as buildings, sidewalks, roads, and medians which constitute the majority of urban areas [4]. Studies tend to focus on urban habitats that mimic more natural habitats, such as domestic gardens [13], brownfield sites [14], parks [15], [16] and forest patches [17]. Understanding the ecology of more highly developed habitats – whether the tops of buildings, sidewalks or street medians – is more difficult. In these places, natural processes interact with active management in ways that may or may not reconcile well with ecological models developed for oceanic islands and forest fragments in “seas” of agriculture [18].

It is likely that aside from observations of rats and roaches in buildings, the little patches of grass and trees in street medians are where urbanites are most likely to see “nature,” whether in the form of a nesting bird or foraging ants. A connection to nature has been hypothesized to serve important functions for psychological and emotional well-being of people in general, and urbanites in particular [e.g. 19], [20], [21]. However, little to nothing is known about the ecology of medians as green patches, areas that if not quite islands are at least visually isolated. If medians are able to conserve a diversity of native species and thereby connect urbanites to nature, that effect will have broad implications [22], [23], [24]. Conversely, if these communities are depauperate wastelands, a relatively barren nature will be the kind experienced by most urbanites. If we can understand what facilitates survival of native species in the most impacted and frequently experienced of green habitats, we can understand how to maintain diversity in other, more ‘natural’ urban habitats.

Among the taxa best able to persist in highly managed urban environments are a subset of arthropods, particularly common insects such as ants. Ants are found in nearly all urban habitats (from woodlands to kitchens and picnics), can be diverse in urban settings [25], [26], [27], are conspicuous, and can have strong ecological [28], [29] and economic impacts [30]. In natural habitats, ant species can function as predators, prey, detritivores, seed dispersers, and herbivores [29], [31]. In urban habitats, ants are likely to also play an important role in scavenging and cleaning cities of human detritus and waste [32]. Where ants are rare, it seems possible that carrion and feces may even be slower to decompose and more likely to accumulate. Ants have been successfully used as indicators of the ecosystem health of different agricultural practices, as well as of the environmental change that such practices inadvertently bring [33], [34], [35]. Ants might also provide a measure of the richness and types of ecological interactions to which urban humans are exposed.

Here we had two objectives which we achieved by sampling 44 medians along Broadway, Park Avenue, and the West Side Highway on Manhattan Island of New York City. First, we sought to characterize the ant community of these “greenways” of one of the most urbanized cities in North America. The New York City metropolitan area is home to around 21 million people. The city alone houses more than 8 million people [3]. Second, within this highly managed environment, we sought to understand how different characteristics of street medians influence the species richness and composition of ants. We test each of a series of potential correlates of both ant species richness and the abundance of two key introduced ant species, *Tetramorium caespitum* and *Nylanderia flavipes*, both of which are known to be present in New York City.

Theory developed in natural settings predicts that larger medians should have more species [36], [37], [38], [39] as should medians with greater vegetative complexity [40], [41], [42]. On the other hand, in urban environment, unique aspects of the managed environment might prove more important. For example, the presence of garbage bins, disturbance, and availability of potential nesting sites (concrete structures and dead wood) might disproportionately influence ant communities [32], [43]. Both of these possibilities assume that ants respond to medians as “islands” of forest-like habitat in a sea of inhospitable city. Medians may be islands for species that forage only in green spaces. On the other hand, some species may forage and live in both medians and the surrounding cement and/or patches of green life even smaller than the medians, such as plants in sidewalk cracks. If many species forage both in medians and between them, ant species richness may be independent of attributes of medians and instead be invariant across replicates. The two focal introduced ant species might respond either to natural or anthropogenic features of medians. Studies of invasive ants tend to emphasize the extent to which they are facilitated by disturbance [44], but whether this is true within cities is ambiguous [45].

As an additional measure of the composition of the median ant faunas, we tested whether depauperate medians tended to have a nested subset of species found at more diverse medians.

## Methods

### Study locations

**Figure 1:**
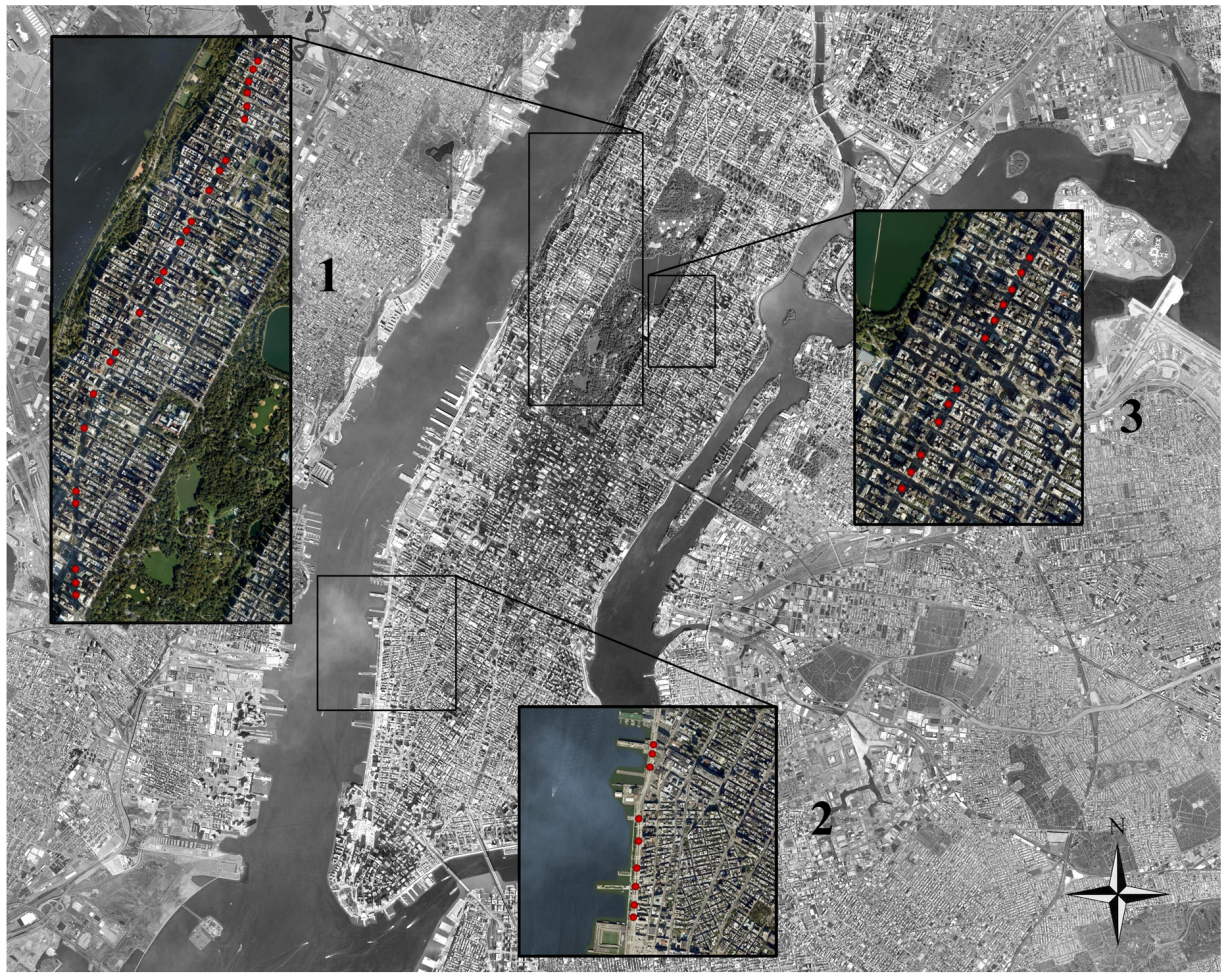
Locations of the 44 street medians included in this study. 1 – Broadway, 2 – West Side Highway, 3 – Park Avenue.

The 44 medians included in this study were located on three major avenues in New York City (Figure 1). These medians were Broadway (23 medians), Park Avenue (12 medians), and West Side Highway (nine medians, WSH in further text). Each of these avenues includes medians that are surrounded by roads on all four sides. The avenues are sufficiently far apart as to preclude frequent dispersal of ants between them, and the medians are sufficiently separated from each other so as to sample independent colonies (i.e. colonies are not foraging from one median to another).

### Ant sampling

Sampling was carried out between 12 June and 28 July 2006. Five unbaited pitfall traps were placed inside each median, three of which were randomly chosen for inclusion in the study and subsequent sorting; this design accommodated occasional trap disturbances by natural or human means. Pitfall traps were plastic drinking cups (7×9 cm) filled with propylene glycol car antifreeze as a killing agent [46]. Pitfall traps sample a subset of all ant species, but are a useful measure of the ants foraging on the ground-surface [46], which are those that human New Yorkers are also likely to see. Since all of the medians included in this study were rectangular, the traps were placed in a manner that ensured that each third of the median contained at least one trap. Traps were emptied after four days, and all the specimens were preserved in 75% ethyl alcohol. After setting the traps, free-roaming ants were also hand sampled using an aspirator for three minutes on each of three locations inside each median to collect any that were in sight, for a total of nine minutes of active hand collecting in each median. All ants collected were preserved in 75% ethyl alcohol. A total of 65 medians were initially sampled, 21 of which had to be excluded from the study because more than two of the traps were removed from them by cleaning crews, stepped on or filled with mowed grass. During laboratory analysis of the traps collected from the 44 remaining medians, ants were separated from non-ant arthropods, identified to species and counted. Ant specimens were deposited in the collection of R. R. Dunn in the Department of Biology at North Carolina State University.

### Environmental variables

Variables measured across each median included both natural and anthropogenic variables likely to influence invertebrate communities. Natural environmental variables included: the number of trees higher than 2 m, percent of canopy cover above 2 m, percent of canopy cover below 2 m, presence of dead wood, non-ant arthropod abundance, and the number of plant species. Anthropogenic variables were the percent of ground covered by ground-covering plants (in horticulture, low-lying plants that are used to cover the ground, and that typically propagate by running rhizomes and/or stolons), number of subway vents (each of which was approximately 6 m by 4 m), number of garbage bins, percent of ground covered by mulch, and number of concrete structures (including man-holes, fire hydrants, and electrical fuse boxes). Each of these variables was used either because previous studies have found them to have an effect on arthropod community species richness [13], [27], [47], [48], [49], or because they were found to be prominent and potentially important features of the medians during the sampling period (as with the number of subway vents and garbage bins).

When counting the number of plant species, only perennials were included because most “seasonal” plants were actively replaced on a monthly basis (Pecarevic personal communication with maintenance crews). The richness of both plants and ants was measured as species density, in other words the total number of species sampled in a given median. We used Chao2 and ICE (Incidence-based coverage estimator) as species richness estimators to estimate the total number of species that would have been sampled had sampling gone to completion both overall and for each of the sets of the three sets of medians considered [76]. Dead wood such as logs and tree stumps were recorded as either present or absent from a median.

Percent tree cover was estimated as the proportion of a median that included trees when seen from one side. All variables measured as percentages (mulch cover, ground covering plants, and canopy cover below 2 m) were estimated in the same manner.

### Data analysis

#### Species richness models

Ordinary least squares regression was used to consider the correlates of ant species richness among medians. In all models, street (Broadway, Park Avenue, or WSH) was included as a variable, to account for differences among medians due to the circumstances associated with each street, their management regime, or other unmeasured factors. *A priori*, the spatial effects due to within median differences seemed the most likely to influence patterns in ant richness and composition. However, to test for additional spatial effects, such as might be associated with spatial autocorrelation in unmeasured independent variables, we tested for spatial autocorrelation in the residuals from the best model within each median. No significant autocorrelation was detected within any street and so we do not consider these spatial effects further. Prior to analysis, all continuous variables were log-transformed to better conform to the normality assumptions of parametric tests, with the exception of those expressed as percentages which were arcsine square root transformed, again for the purpose of normalizing the data [50]. All statistical analyses were done using JMP [51].

We began with a model including all of the variables of interest (number of trees higher than 2 m, percent of canopy cover above 2 m, percent of canopy cover below 2 m, presence of dead wood, the number of plant species, percent of ground covered by ground-covering plants, number of subway vents, number of garbage bins, percent of ground covered by mulch, and number of concrete structures). We then simplified the model by removing non-significant variables (α = 0.05) in a stepwise fashion [52], to explore the relative contributions of the various terms included in the full models. Although stepwise simplification approaches have been criticized [53], there is no perfect approach to choosing among multiple predictor variables in the absence of prior models [54]. We interpret our “best model” as being the best model in terms of accounting statistically for variation in ant species richness, though we acknowledge that causal mechanisms may be more complicated. As such, we present the correlation matrixes for pairwise comparisons among all possible variables in the supplement (Figure S1).

#### Nestedness

In habitats that include disturbance, low richness sites often include a nested subset of those species in high richness sites. At the opposite extreme, low richness sites might contain a unique subset of species not found in more diverse sites. Our species by site data were tested for nestedness using the Nestedness Calculator [55] available at a website from the Field Museum in Chicago (http://www.fieldmuseum.org/research_collections/zoology/nested.htm). This program measures the extent of the order present in nested presence-absence matrices. A perfectly ordered or nested system, absent of all randomness, is characterized as maximally “cold” (0°), whereas a system absent of all order may be labeled as maximally “hot” (100°) [56]. A Monte Carlo simulation was used to test whether the data were significantly different from a random sample. The matrix was randomized 500 times. The measurements of temperature from the observed data were then compared to the distribution of values from the randomized matrices.

## Results

### Ant community composition

A total of 6,619 individual ants of 13 species and 11 genera was recorded from the street medians of Manhattan (Table 1). Species richness estimators indicate that were sampling to have gone to completion 12 species would have been detected on Broadway, 8 on Park Avenue, 12 on the West Side Highway, and 14 overall. The three numerically most dominant species (Table 1) accounted for most of the individual ants sampled (94%). These were the Pavement ant (*Tetramorium caespitum*), the Thief ant (*Solenopsis molesta*), and the Cornfield ant (*Lasius neoniger*). These were also the three species found at the most sites. All three of these species are frequently encountered in urban and suburban environments across a broad geographic area [48], [57], [58]. In addition to these three species, *Nylanderia flavipes* [59] was also very frequently sampled, although not numerically dominant. Of the four most common species, two (*L. neoniger* and *S. molesta*) are native, and two (*T. caespitum* and *N. flavipes*) are introduced (Table 1). Both native and introduced ant species coexist in Manhattan in relatively large numbers. Although the most diverse and least diverse medians sampled (eight and two species, respectively) represented a large range, most medians were similar in their ant species richness. In fact, 31 of the 44 medians had between three and five ant species.

**Table 1:** Ant species found in New York City medians.

Species	No. of medians collected	% of medians collected	Number of individuals	% of total number of individuals	Status
*Tetramorium caespitum*	41	93.2%	3484	52.6%	Introduced
*Lasius neoniger*	39	88.6%	1695	25.6%	Native
*Solenopsis molesta*	28	63.6%	1022	15.4%	Native
*Nylanderia flavipes*	23	52.3%	195	3.0%	Introduced
*Camponotus pennsylvanicus*	7	15.9%	17	0.3%	Native
*Lasius alienus*	5	11.4%	40	0.6%	Native
*Brachymyrmex depilis*	4	9.1%	10	0.2%	Native
*Formica* subsericea	4	9.1%	57	0.9%	Native
*Monomorium minimum*	4	9.1%	38	0.6%	Native
*Pheidole tysoni*	4	9.1%	40	0.6%	Native
*Aphaenogaster rudis*	2	4.6%	4	0.1%	Native
*Formica* sp. II	2	4.6%	3	0.1%	Native
*Pachycondyla chinensis*	1	2.3%	4	0.1%	Introduced

### Correlates of ant abundance and species richness

**Figure 2:**
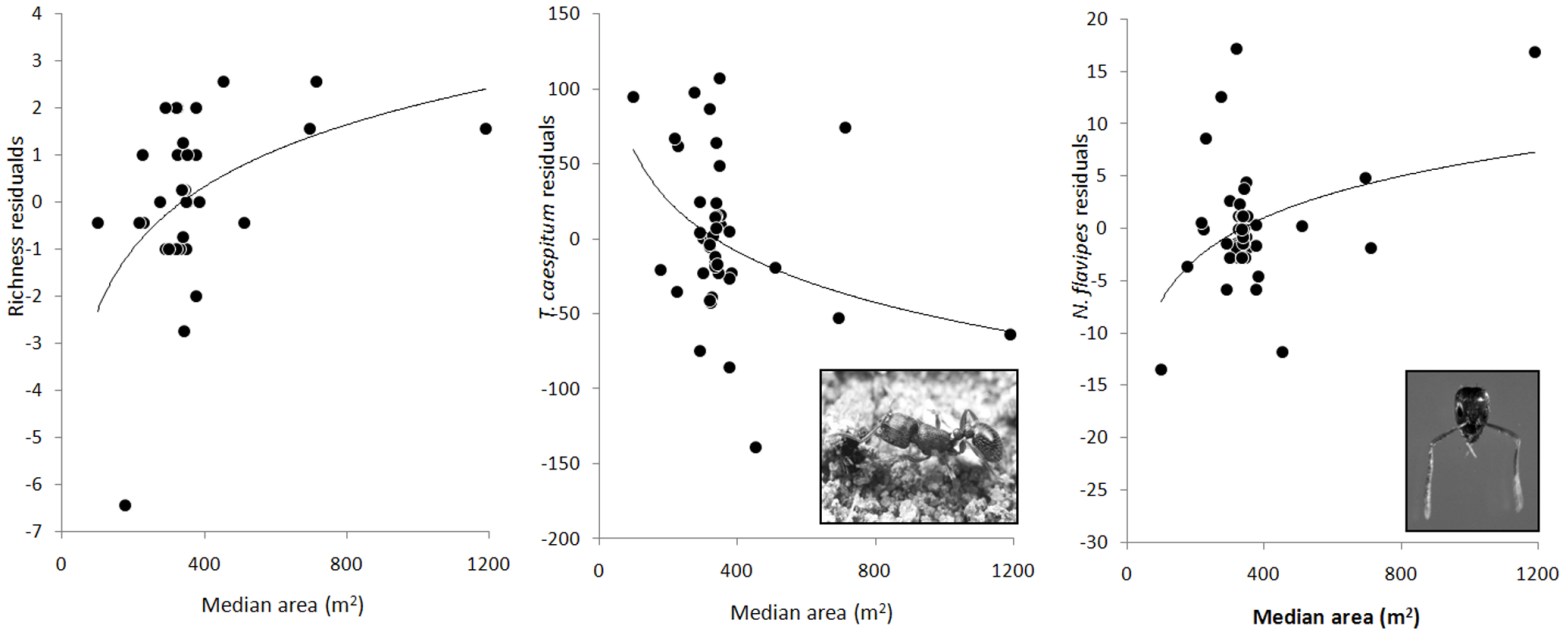
The relationship between ant species richness, *T. caespitum* abundance and *N. flavipes* abundance and area after we accounted for other variables significant in the final models. Species richness and *N. flavipes* abundance increase with area, whereas the abundance of *T. caespitum* declines.

Given the similarity among medians in their ant species composition and richness, relatively little variation was left to be explained by possible biotic and abiotic correlates. In stepwise regression, the only significant factor in the models was the street on which the medians were located. Once “street,” was accounted for, the only other variable that improved the model was the area of the median, with larger medians having more species (Table 2, Figure 2). As our approach here is correlational, it begs for a second, more experimental step to disentangle the mechanistic basis of the correlation of richness with area.

**Table 2:** Correlates of ant species richness, where each site (N = 44) is an individual street median.

	SSE	DF	MSE	R^2^
**Full model**	87.4	41	2.1	0.47

	Est.	SS	F-Ratio	P
**Log (Area)**	1.98	21.5	10.1	0.0028
**Street (West Side Highway - Broadway & Park Ave)**	−1.51	64.8	30.4	<0.00001

### Correlates of introduced species abundance

The abundance of the introduced ant species *T. caespitum* was negatively correlated with tree density. Medians that were less forest-like had more *T. caespitum* (Table 3). The other abundant introduced ant species *N. flavipes*, on the other hand, was most abundant at the medians that were more forest-like, increasing when a larger proportion of the median was tree covered (Table 4).

**Table 3:** Correlates of the abundance of the pavement ant, *Tetramorium caespitum*.

	SSE	DF	MSE	R^2^
**Full model**	93772.9	39	2404.4	0.59

	Est.	SS	F-Ratio	P
**Log (Edge/Area)**	142.2	14499.7	6.03	0.02
**Street (Broadway & Park Ave - West Side Highway)**	−56.3	63138	13.1	<0.00001
**Street (Park Ave - Broadway)**	−28.0	22823.2	9.5	0.004
**Canopy <2 meters**	−0.78	18012.7	7.5	0.009

**Table 4:** Correlates of the abundance of the introduced ant, *Nylanderia flavipes*.

	SSE	DF	MSE	R^2^
**Full model**	1099	39	28.2	0.50

	Est.	SS	F-Ratio	P
**Log (Area)**	0.02	268.9	9.5	0.004
**Street (Broadway & Park Ave - West Side Highway)**	–5.7	458.9	8.2	0.001
**Canopy >2 meters**	8.07	69.2	2.5	0.03

### Nestedness

Using the Nestedness Calculator [55] the temperature of the ant fauna of Manhattan medians was found to be 11.97° out of a possible 100°, in other words strongly nested. After randomizing the matrix 500 times, average temperature of the system was found to be 55.21° (S.D. = 5.12°), using the Monte Carlo test. The probability that temperature below 11.97° will be drawn was P = 1.8×10^−17^. It is therefore safe to conclude that the species inhabiting the medians are very nested. Species poor sites contain a subset of those species found at species rich sites.

## Discussion

In perhaps the most often viewed green space in the world, the medians of New York City streets, we found thirteen different species of ants and evidence that even more species are likely to occur. The species collected included subterranean species that tend aphids, much in the way that humans tend cattle (*Lasius neoniger*), a human commensal species (*Tetramorium caespitum*) that appears to nest preferentially under cement, and a keystone, seed-dispersing forest species (*Aphaenogaster rudis*). The most abundant species (*Tetramorium caespitum*) is introduced, but the second most abundant species (*Lasius neoniger*) and the majority of species overall were native. On Broadway, species native to North America coexist alongside those native to other regions of the world. Manhattan is, if not quiet a melting pot of ant species, at least a mixing bowl.

In contrast to the case for many natural ecosystems [60], [61], [62], few of the environmental variables we considered were significantly correlated with the species richness of ants in medians. Why some medians had more ant species than did others remains enigmatic. What is clear is that the species assemblages were strongly nested, i.e. the primary differences in species richness from one median to the next was due to the presence of rare species in the most diverse medians. While it is unclear from our analyses what allows rarer species we sampled to persist in medians, it is clear they do. Some of these species may be more common than is apparent given our sampling method. Future studies that employ additional forms of sampling that catch arboreal, soil and litter dwelling ants, are likely to yield more species than we were able to detect. Even those species we collected deserve additional study in a natural history, ecological or evolutionary context. For example, a median on West Side Highway close to the Hudson River has *Pheidole tysoni* living on it. This species can be found within a walking distance of several million people and is geographically widespread, but like most insect species, even its most basic biology is poorly known.

Introduced ant species are often described as being favored by disturbance [15]. In New York City, we found patterns in introduced ant species abundance to be more complex. The four ubiquitous species (*T. caespitum*, *L. neoniger*, *S. molesta*, and *N. flavipes*) were found to coexist on almost all of the medians observed. In contrast, the pavement ant, *T. caespitum*, was most abundant in small medians with the greatest edge to area ratio, and particularly if those medians had few trees. This is consistent with a study of urban ants carried out in San Francisco [73], where this species was found only in parks where the dominant vegetation type was herbaceous. *Nylanderia flavipes*, on the other hand, was most abundant in the largest medians, particularly if they had more trees. These results reconcile well with observations of the natural history of these species, which are often thought of as pavement (*T. caespitum* [48], [63]) and forest (*N. flavipes* [25], [64]) specialists. *Nylanderia flavipes* is Asian, potentially Japanese, in origin and has spread to the United States via imported plant material [64], [65]. This species nests in leaf-litter, rotten wood, and in the soil of grasslands and forests [15], a natural history that reconciles well with our observation that tree cover was a predictor of the abundance of this species in medians. *T. caespitum* is considered a human commensal because of its close association with humans [66], [67]. It often nests under bricks and in sidewalk crevices [48]. *T. caespitum* is highly territorial, omnivorous, and has a foul taste (at least to myrmecologists) which makes it unpalatable to predators [66], [68], [69], all of which may predispose it to life in cities, though why it does better where more cement is present, both in New York and elsewhere, remains unknown.

Four individuals of the Asian Needle Ant (*Pachycondyla chinensis*) were discovered during this survey. This ant has recently been recognized as a public health threat in the southeastern United States (South Carolina, Georgia, North Carolina, and Virginia) [70], [77], but it had not been identified in the state of New York until the sampling done during this study [71]. In some parts of North Carolina, this species is now the most common ant (70 – 80% of all individuals encountered) where it is present [72]. The great potential abundance of this species, combined with the high incidence with which its stings cause anaphylactic shock, suggests the appearance of this species in New York State and particularly in densely populated Manhattan should be taken seriously. In the late spring of 2007, an additional survey of the leaf-litter in the median in which *P. chinensis* was collected was carried out, but no additional individuals were captured. Additional studies in Manhattan, and New York more generally, are necessary to understand if the individuals we sampled were a nascent and unsuccessful propagule from the south or part of the expanding range of this species. It is interesting to note that in a study carried out by Yamaguchi [15], *P. chinensis* was found inhabiting the same area as *Nylanderia flavipes* in their native Japan, as is also apparently the case in New York City.

While the species richness within the medians studied here was relatively high compared to less human dominated habitats, it is notable that many of the species we encountered were similar (though importantly not identical) to those found in other North American cities, such that a relatively small subset of ant species probably represent most of the encounters humans have with ants in much of the United States and Canada. In the seven studies of urban habitats in North American cities of which we are aware [16], [17], [28], [48], [57], [73], all but one of the studies [28] shared some species with those found in our study. Even in a study of San Diego, California, where the surrounding natural habitat is semi-desert rather than temperate forest (Table 5), many species were shared. Additionally, two of the most abundant species in New York were also among the most abundant species in two other cities. Whatever effects these species are having on urban ecosystems, they are carrying them out across a very wide geographic range.

**Table 5:** Comparison of the species most frequently sampled in this study, with those in other urban ant studies.

Reference	Region	Distance from Manhattan, NY (km)	Total Species	Most abundant species	Shared species (with this study)	Notes
This study	Manhattan, NY, USA	0	14	***T. caespitum*** **, ** ***L. neoniger*** **, ** ***S. molesta*** **, ** ***N. flavipes***	13/13	
Danoff-Burg & Melnick (2004)	Manhattan, New York City, USA	0	24	***N. flavipes*** **,** *Prenolepis imparis, Ponera pennsylvanica, * ***L. neoniger*** **,** *L. alienus, Aphaenogaster rudis*	7/13	Unpublished data
Nuhn & Wright (1979)	Raleigh, NC, USA	650	59	*Pheidole vinlandica, Forelius pruinosus, Monomorium minimum, * ***T. caespitum*** **, ** ***L. neoniger*** **,** *Nylanderia sp. parvula* complex, *Aphaenogaster spp.*	10/13	Habitat surveyed was the North Carolina State University Campus in Raleigh
Lessard & Buddle (2005)	Quebec, Canada	715	24	*Aphaenogaster sp., Camponotus pennsylvanicus, Formica glacialis, L. alienus, * ***L. neoniger*** **, ** ***T. caespitum***	6/13	Only the urban aspect of this study
Thompson & McLachlan (2007)	Manitoba, Canada	2000	10	*C. pennsylvanicus, F. glacialis, Lasius pallitarsis*	1/13	Only the urban aspect of this study
Sanford et al (2009)	Lake Tahoe basin, USA	3800	42	*Formica sybilla, Formica lassioides, Myrmica tahoensis, Camponotus modoc, Formica accreta, Camponotus vicinus, T. sessile*	0/13	Only the most urbanized of the sites characterized by this study.
Suarez et al (1998)	Coastal southernCalifornia	4000	46	*L. humile, Dorymyrmex insanus, Prenolepis imparis, Solenopsis molesta, Leptothorax andrei*	2/13	
Clarke et al (2008)	San Francisco	4600	15	*Linipithema humile, Temnothorax andrei, Monomorium ergatogyna, T. sessile*	2/13	

The number of shared species is in some cases an estimate as in some studies certain ant types were only identified to the genus level. For studies that had such separation of results, only the urban aspect was considered. Shared species indicates the number of species found in this study that were also found in each of the other studies, where the maximum would be 13/13.

The rare species in Manhattan, as in other cities need to be studied in order to understand how they persist and how we might favor their continued persistence. Yet, the more successful and common species may be the ones from which we have the most to learn. Recently, the origins [74] and life history of urban populations of *Tapinoma sessile* were considered. Menke et al. [74] together with Buczkowski [75] found that many or even most urban colonies of *T. sessile* have life history traits that are absent in colonies living in natural habitats (multiple queens and large colonies in particular). These life history traits have arisen independently in different lineages of the species in association with the transition to city life [74]. Whether other urban species have also made these convergent shifts is unknown. While there are many ways to be a New Yorker, it may be that there are fewer ways to be an urban ant.

## Supporting Information

Figure S1 The correlation and scatter plot matrix for pairwise comparisons among all possible variables. (% high canopy  =  percent of canopy cover above 2 m, % mulch  =  percent of ground covered by mulch, % high canopy  =  percent of canopy cover below 2 m, percent of ground covered by ground-covering plants, trees>2 m  =  number of trees higher than 2 m, plant sp. =  number of plant species)
(1.73 MB TIF)
